# Hearing Impairment Among Nursing Students: A hearWHO-Based Screening Study

**DOI:** 10.7759/cureus.98520

**Published:** 2025-12-05

**Authors:** Bhagyashree Kathari, Balu PS, Kamal Pandyan, Vanamala Sathish

**Affiliations:** 1 Community Medicine, Subbaiah Institute of Medical Sciences and Research Centre, Shivamogga, IND; 2 Centre for AI Research and E-Health, Subbaiah Research Institute, Shivamogga, IND; 3 Otorhinolaryngology, Subbaiah Institute of Medical Sciences and Research Centre, Shivamogga, IND; 4 Nursing, Tadikela Subbaiah College of Nursing, Shivamogga, IND

**Keywords:** headphone use, hearing assessment, hearwho, nursing student, hearing loss

## Abstract

Background

Hearing loss among young adults is increasingly recognized as a public-health concern, often linked to harmful listening practices. While conventional audiometry is the diagnostic standard, smartphone-based screening tools such as the WHO’s hearWHO offer a low-cost, scalable option for early detection. This study estimated the prevalence of possible hearing impairment in a cohort of nursing students using the hearWHO app and examined related risk factors.

Methods

In this cross-sectional study, 592 nursing students (≥18 years) at Subbaiah Institute of Nursing, Shivamogga, underwent hearing screening with the hearWHO Digits-in-Noise test. Participants who recorded scores below 50 dB were retested after 15 minutes, and the mean of the two readings was used for final classification. A pretested questionnaire collected demographic data, headphone habits, and relevant medical history. Data were analyzed in IBM SPSS Statistics for Windows, Version 20 (Released 2011; IBM Corp., Armonk, New York, United States); significance was set at p < 0.05.

Results

The sample had a mean age of 20.5 ± 1.5 years and comprised 84.8% females. On initial testing, 475 participants (80.2%) scored 50-75 dB, 102 (17.2%) scored below 50 dB, and 15 (2.5%) scored above 75 dB. After reassessment, the prevalence of hearing impairment (mean score <50 dB) was 87 students (14.6%) (p = 0.000). Significant associations with poorer hearWHO scores were observed for age distribution (p = 0.018), type of headphones used (p = 0.045), and noncompliance with device volume alerts (p = 0.001). The average daily headphone use (2 ± 1.5 hours) showed no significant correlation with hearing thresholds (r = −0.009, p = 0.869).

Conclusions

The hearWHO application is a practical screening method for identifying potential hearing impairment in a student population. Behavioral factors related to listening practices, especially headphone type and disregard of volume alerts, were more closely linked to impaired screening results than either demographic variables or daily listening duration.

## Introduction

Hearing loss is the partial or complete inability to hear sounds. It can severely affect communication, learning, and social interaction [1]. Hearing loss has been increasing steadily in the global population. The WHO estimates that by 2050, over 700 million people, about one in ten, will experience disabling hearing loss. The economic impact will approach US$1 trillion each year, showing the high cost of this issue. Furthermore, well over one billion young adults may risk permanent hearing damage from unsafe listening habits. This situation stresses the urgent need for preventive actions and public health measures [2].

The National Programme for Prevention and Control of Deafness (NPPCD) was launched in the year 2008 in India. The aim of this program is to reduce preventable causes of hearing loss and reduce the prevalence of deafness to below 1% by 2030 at the national level [3]. To achieve this target, we crucially need reliable data on the prevalence of hearing problems and their contributing factors.

Advancements in mobile health technologies have brought about innovative strategies for hearing screening. Among these, the WHO’s “hearWHO” smartphone-based application is gaining attention, as it is cost-effective and can be employed even without audiological resources, since it does not require trained personnel [4]. Considering its potential, ample evidence regarding its utilization in India exists among nursing students, one of the major populations often exposed to headphone use and other risk factors. Therefore, this study was undertaken, which aimed to estimate the prevalence of impaired hearing among nursing students using the hearWHO app and identify behavioral and demographic factors associated with impaired hearing. The primary objective of our study was to assess the prevalence of hearing impairment among nursing students using the hearWHO screening tool. A secondary objective was to identify factors associated with lower hearWHO scores.

## Materials and methods

Study setting and duration

The research was carried out at the Subbaiah Institute of Nursing College, Shivamogga, over the designated study period.

Study design

This was a cross-sectional investigation designed to measure the prevalence of hearing impairment among nursing students using a smartphone-based application and to evaluate associated risk factors, including headphone usage.

Study population

All nursing students aged 18 and over enrolled at the institution were considered eligible.

Sampling technique

A universal sampling approach was applied. Every student who met the eligibility criteria and consented to participate was included in the study.

Sample size

The sample size was determined using a formula for estimating population proportion with a 95% confidence interval and an allowable error of 2.5%. Mogan et al.'s prior study [5] reported a prevalence of hearing impairment of 9.4%, which served as the basis for the calculation. This method produced a minimum required sample of 525 participants. Accounting for a potential 10% nonresponse rate, the target sample size was adjusted to 584.

Inclusion and exclusion criteria

The study included nursing students aged 18 years or older who provided written informed consent to participate. Students with a previously diagnosed hearing impairment or those currently using hearing aids were excluded from the study. Additionally, students who were absent during the data collection period or declined to participate were also excluded.

Hearing Screening Procedure

Hearing assessment was performed using the hearWHO mobile application developed by the WHO [4]. The application is based on a digits-in-noise (DIN) testing method, where participants are asked to recognize sets of three spoken digits presented against background noise at varying levels.

All screenings were conducted in a quiet, designated classroom to reduce ambient noise. A uniform over-the-ear headphone was provided for every participant to minimize variability. Students were briefed on the process and instructed to adjust the volume to a comfortable level before testing began. The app generated a signal-to-noise ratio (SNR) score reflecting hearing ability. Scores were interpreted as follows: 75 dB, normal hearing; 50-75 dB, hearing may be affected; monitoring recommended; <50 dB, possible hearing impairment; further assessment advised.

Ambient noise was minimized by conducting the screening in a quiet room with controlled surroundings. All participants used the same smartphone model with a standardized volume setting as per the hearWHO recommendations to ensure consistent device calibration.

Participants with scores <50 dB were retested after 15 minutes, and the mean of both scores was used for classification. This 15-minute interval was chosen as recommended by the hearWHO guidelines to minimize temporary auditory fatigue and environmental influence before retesting.

Data collection on risk factors

A structured, pretested questionnaire was administered to gather information on age and gender; exposure to loud sounds in occupational or recreational settings; type, frequency, and duration of personal audio device use; and history of ear infections, trauma, or ENT consultations.

Statistical analysis

Data from the hearWHO app and questionnaires were entered into MS Excel (Microsoft Corporation, Redmond, Washington, United States) and analyzed using IBM SPSS Statistics for Windows, Version 20 (Released 2011; IBM Corp., Armonk, New York, United States). Descriptive statistics (frequencies, percentages, means, and standard deviations) summarized participant characteristics and hearing scores. The prevalence of hearing impairment was determined by the percentage of students whose final scores were less than 50 dB. Associations between hearing status and potential risk factors (age, gender, headphone habits, etc.) were tested using the chi-square test for categorical variables. A p-value < 0.05 was considered statistically significant.

Ethical considerations

Ethical approval was obtained from the Institutional Ethics Committee of SUIMS, Shivamogga (Ref: IEC-SUIMS/156/2025-26). Written informed consent was obtained from all participants. Anonymity and confidentiality were ensured. Students identified with possible hearing impairment (hearWHO score <50 dB) were informed and referred for further ENT evaluation.

## Results

A total of 592 nursing students were screened for hearing impairment using the hearWHO application, and the mean age of the participants was 20.5+_1.5 years, ranging from 17 to 36 years. The majority (502, 84.8%) were female participants, with a sex ratio of 1:5.6, and male participants of 90 (15.2%) (Table 1).

**Table 1:** Sociodemographic details of the study participants (N = 592)

Sociodemographic parameters	N (%)
Age in years	
15-20	308 (52)
21-25	279 (47.1)
26-30	5 (0.8)
Gender	
Male	90 (15.2)
Female	502 (84.8)

hearWHO grading

**Figure 1:**
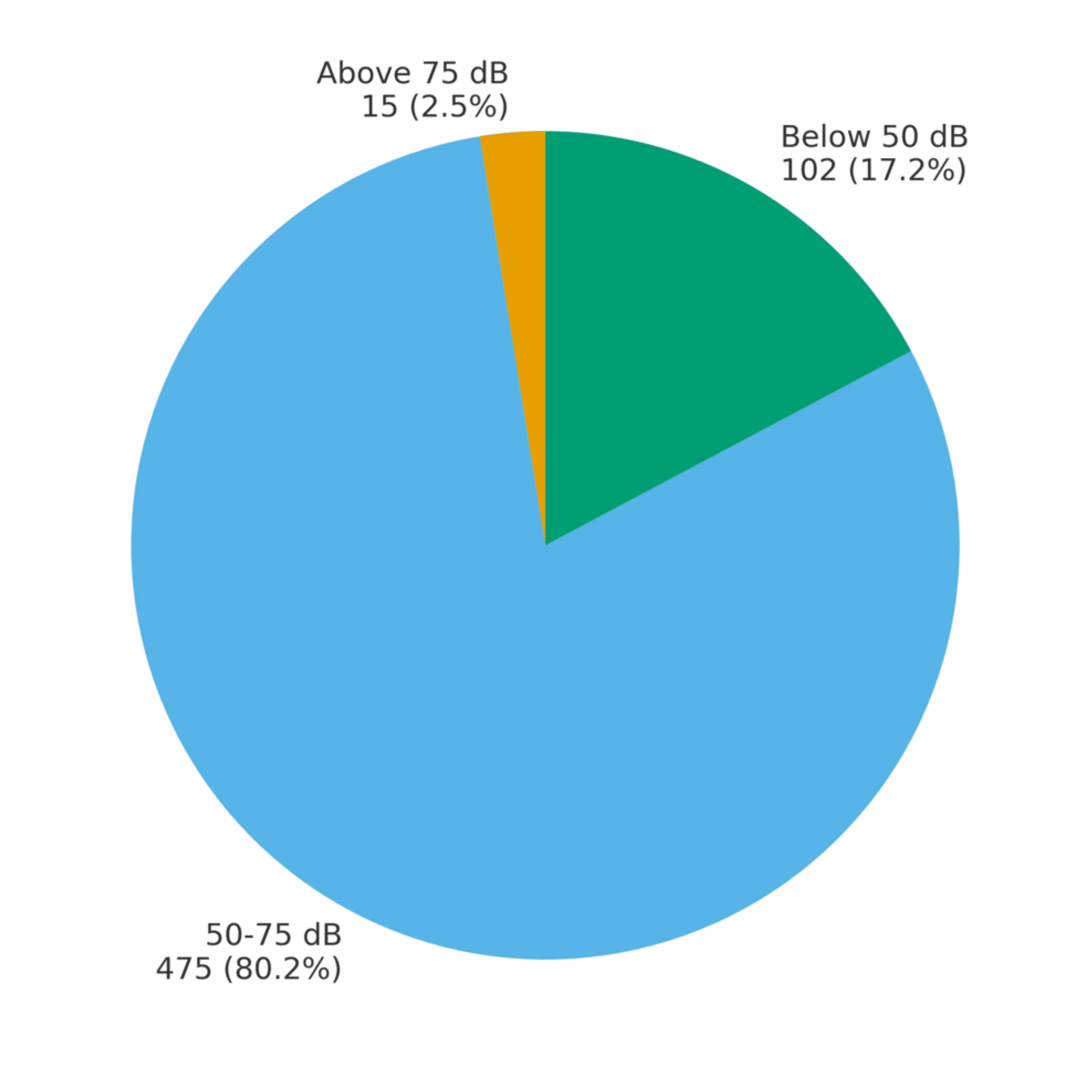
Hearing impairment grading as per the hearWHO app among nursing students

Among the 592 nursing students screened, the majority (475 students, 80.2%) had hearing thresholds between 50 and 75 dB (mean 52.64 ± 5.5 dB), indicating the need for further monitoring, while 102 (17.2%) recorded values below 50 dB (mean 39.09 ± 0.9 dB), raising concerns of possible hearing loss. After reassessing these students 15 minutes later, the average of both readings was used for classification, and the overall prevalence of hearing impairment was found to be 87 (14.6%), which was statistically significant (p < 0.05). Only 15 students (2.5%) demonstrated thresholds above 75 dB (mean 80.67 ± 2.5 dB) (Figure 1, Table 2).

**Table 2:** Distribution of the hearWHO grading among nursing students (N = 592) ANOVA: analysis of variance; *One-way ANOVA test with F value: 531.60

Hearing category	N (%)		Mean ± SD	Minimum	Maximum	
Above 75 dB	15 (2.5)		80.67 ± 2.5	80	90	
50-75 dB	475 (80.2)		52.64 ± 5.5	50	70	
Below 50 dB	102 (17.2)		39.09 ± 0.9	30	40	
Total	592 (100)		51.16 ± 8.4	30	90	

Age-wise distribution of the study participants and its association with the hearWHO grading

**Figure 2:**
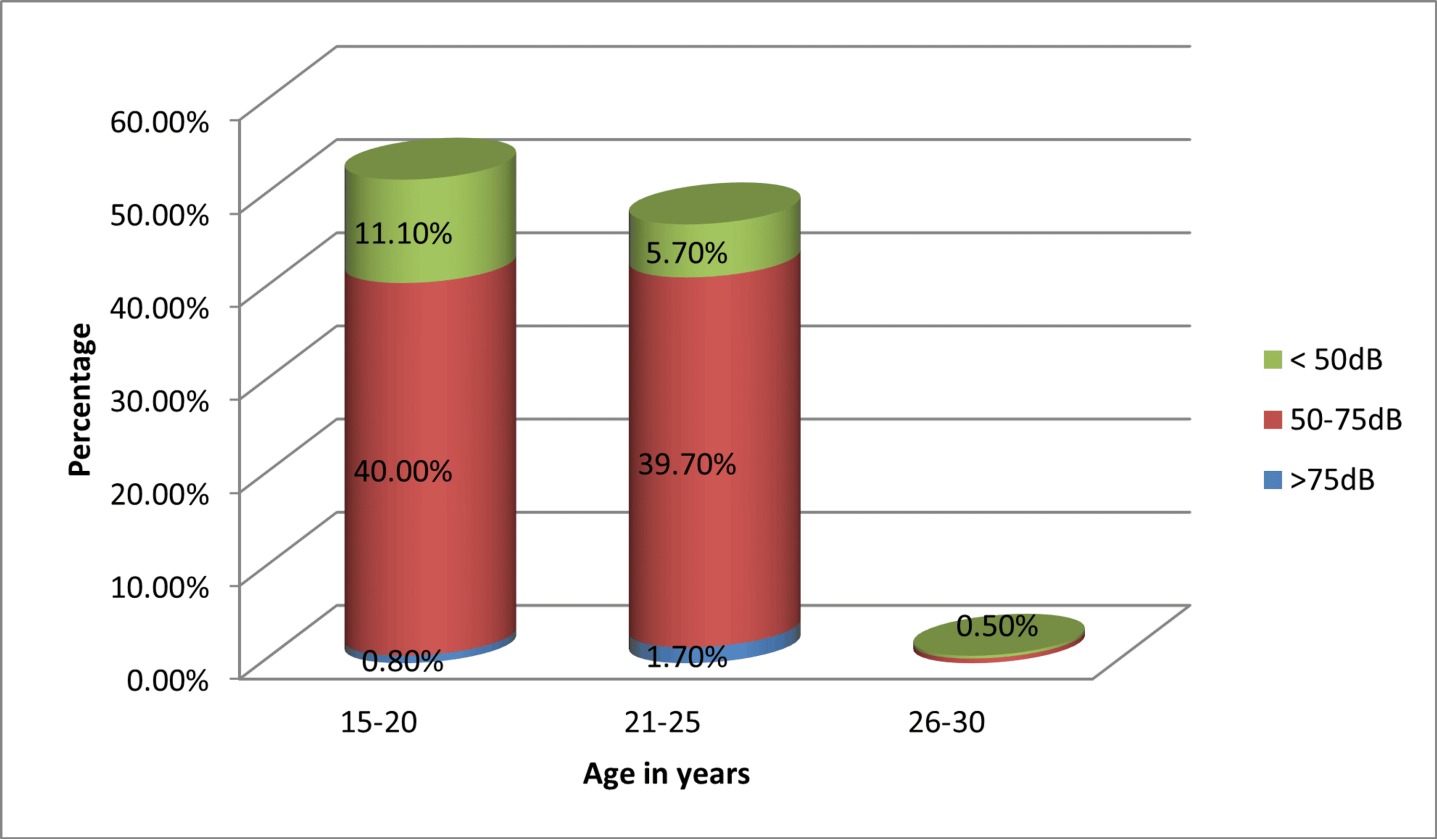
Age-wise distribution of the study participants and its association with the hearWHO grading

Among the nursing students, the largest proportions were in the age groups of 15-20 years (52.2%) and 21-25 years (47.1%), with very few participants older than 25 years. Most students across all age groups had hearing thresholds in the 50-75 dB range, though impaired scores below 50 dB were more frequent in those <20 years (21.4%) compared to the 21-25 years group (12.5%). The observed differences in hearing status across age groups were statistically significant (p = 0.015) (Figure 2).

Association between the hearWHO grading and risk factors for hearing loss

Among the various risk factors assessed, most, including gender, history of screening, family history of hearing loss, headphone usage, ear pain, and current upper respiratory infection, showed no significant association with hearing impairment. However, a significant association was observed with the type of headphone used (p = 0.045), where overhead headphone users had higher impairment compared to earphone users, and with compliance to device volume alerts (p = 0.001), as those ignoring alerts demonstrated greater hearing loss. This suggests that listening practices, rather than demographic or medical history, play a more crucial role in determining hearing outcomes in this population. The mean daily headphone usage was 2 ± 1.5 hours, and no significant correlation was observed with hearing thresholds (r = -0.009, p = 0.869), suggesting that duration of daily headphone use did not influence hearing outcomes in the study population (Table 3).

**Table 3:** Association between the hearWHO grading and risk factors for hearing loss among nursing students *p-value < 0.05 is considered to be statistically significant

Risk factors	hearWHO grading N(%)			Total (592)	Chi-square value	p-value
	>75 dB	50-75 dB	<50 dB			
Gender					0.28	0.866
Male	3(0.5)	72(12.2)	15(2.5)	90(15.2)		
Female	12(2)	403(68.1)	87(14.7)	502(84.8)		
History of screening for hearing impairment					2.07	0.354
Yes	0	36(6.1)	5(0.8)	41(6.9)		
No	15(2.5)	439(74.2)	97(16.4)	551(93.1)		
Family history of hearing loss					1.78	
Yes	0	20(3.4)	2(0.3)	22(3.7)		0.41
No	15(2.5)	455(76.9)	100(16.9)	570(96.3)		
Headphone usage					3.09	0.213
Yes	15(2.5)	424(71.6)	95(16)	534(90.2)		
No	0	51(8.6)	7(1.2)	58(9.8)		
Type of headphone used					6.21	0.045^*^
Overhead	12(2)	300(50.7)	76(12.8)	388(65.5)		
earphones	3(0.5)	175(29.6)	26(4.4)	204(34.5)		
Pain in ears					0.91	0.633
Yes	0	27(4.6)	6(1)	33(5.6)		
No	15(2.5)	448(75.7)	96(16.2)	559(94.4)		
Current history of upper respiratory infection					2.51	0.284
Yes	3(0.5)	66(11.1)	9(1.5)	78(13.2)		
No	12(2)	409(69.1)	93(15.7)	514(86.8)		
Comply with alert to volumes in audio devices					13.74	0.001^*^
Yes	1(0.2)	181(30.6)	53(9)	235(39.7)		
No	14(2.4)	294(49.7)	49(8.3)	357(60.3)		

## Discussion

This study evaluated the hearing status of nursing students using the hearWHO mobile application. The findings highlight the utility of mobile-based screening in early detection of hearing problems among young adults, a group traditionally perceived as low risk but increasingly vulnerable due to unsafe listening practices. Compared to conventional audiometry, mobile health tools are more feasible for large-scale use in educational institutions, particularly in resource-limited settings.

In the present study, 14.6% of participants were classified as having impaired hearing, while the majority scored in the 50-75 dB range, suggesting suboptimal hearing that warrants monitoring. Only a small minority achieved normal scores (>75 dB). These results emphasize the importance of proactive screening in younger populations.

The prevalence reported here is similar to other student-based studies. Mogan et al. in Delhi found that 9.4% of medical undergraduates scored below 50 dB, with more than half falling in the intermediate range [5]. Gupta et al. also reported high rates of subclinical hearing decline among young adults in India, often linked to prolonged headphone use [6]. A Bengaluru study by Ramesh Masthi et al. documented that nearly one-third of professional students screened with hearWHO scored <50 dB [7].

Globally, evidence also points to rising hearing concerns in youth. A systematic review by Keppler et al. found that more than 20% of young adults exposed to loud music through personal listening devices showed measurable hearing threshold shifts [8]. Similarly, Sulaiman et al. in Malaysia demonstrated that students with frequent headphone use had significantly poorer hearing thresholds compared to their peers [9]. A study among US college students by Le Prell et al. reported that almost 25% experienced temporary auditory changes after listening to music at high volumes, raising concerns about long-term effects [10].

In our study, age (<20 years), headphone type, and ignoring device volume alerts were significantly associated with poorer hearing outcomes. These behavioral factors mirror patterns reported elsewhere. For example, Mogan et al. showed that noncompliance with volume warnings increased the likelihood of impaired scores [5], while Keppler et al. emphasized that over-the-ear headphones, particularly when used in noisy environments, increased exposure risks [8]. The lack of association with duration of daily use in our study aligns with earlier findings suggesting that intensity of exposure, rather than duration alone, may be the more critical determinant of risk [9,11,12].

Medical and demographic factors such as gender, family history, and recent respiratory symptoms did not show significant associations in our study. This differs from Garg et al., who reported higher prevalence in those with recurrent ENT problems [13], and Arju et al., who noted associations with clinical histories of infections [14]. Our student population's relatively young age and overall good health status may explain the absence of these links.

The combined results from this and previous studies highlight the potential of hearWHO and similar mHealth tools in promoting hearing health. Their portability, scalability, and acceptance among young adults make them ideal for institutional and community screening [15,16]. Future strategies should not only target older adults, who remain most at risk globally, but also incorporate interventions aimed at younger populations who are increasingly exposed to recreational noise.

Limitations of our study 

The study was conducted in a single nursing institution, which may limit the generalizability of results to other student populations. The hearWHO app is designed as a screening tool; it does not differentiate between conductive and sensorineural hearing loss or provide detailed grading of severity. This study didn't evaluate the formal usability or diagnostic accuracy of hearWHO.

The application is currently available only in English, which may restrict accessibility for non-English-speaking populations. The tool is not intended for use in young children, which limits its applicability across all age groups. As the assessment is based on self-administered responses, the possibility of response bias cannot be excluded. Lastly, we also acknowledge the limitations of the study with respect to device calibration and environmental noise, both of which could have introduced measurement variability.

## Conclusions

The findings of this study indicate that the hearWHO application is a practical and accessible tool for detecting early signs of hearing impairment among young adults. Its ease of use, low cost, and scalability make it well-suited for integration into institutional health programs, particularly in environments with limited audiology resources. Listening behaviors, including the type of headphones used and compliance with device volume alerts, were more strongly linked to impaired hearing than demographic characteristics or duration of headphone use. Regular use of such mobile-based tools can contribute to early identification, awareness, and timely referral, ultimately helping to reduce the burden of hearing loss. The use of a standardized mobile-based tool (hearWHO) increases its feasibility and potential for public health applications.
